# Supplementary material for: Calibrating dimension reduction hyperparameters in the presence of noise
Source: PLoS Comput Biol. 2024 Sep 12;20(9):e1012427. doi: 10.1371/journal.pcbi.1012427 (PMC11421778; doi:10.1371/journal.pcbi.1012427)
Supplement: S1 Appendix — (PDF) [file pcbi.1012427.s001.pdf]

Supporting Information

1 Simulated Examples

1.1 Trefoil Plots

For the trefoil example, the signal Y consisted of a trefoil knot embedded in three dimensions containing 500 points. $Z + \epsilon$ was constructed by adding seven superfluous dimensions and isotropic Gaussian noise. Various degrees of noise were tested ($sd = 5, 10, 15, 20, 25, 30$). The first two plots depict Trustworthiness vs. Perplexity and the trustworthiness-maximizing embeddings for the $sd = 10$ case. The third plot shows the trustworthiness-maximizing perplexity for the different degrees of noise.

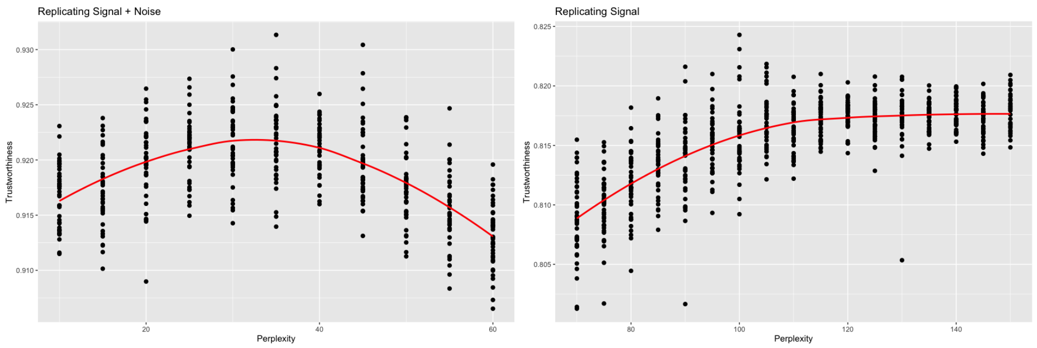

Fig A. Trustworthiness vs. perplexity for sd = 10 (trefoil). t-SNE outputs were calculated with varying perplexities. Local performance was measured via trustworthiness. The trustworthiness-maximizing perplexity was 35 when comparing against the original data, while the trustworthiness-maximizing perplexity was 100 when comparing against just the signal.

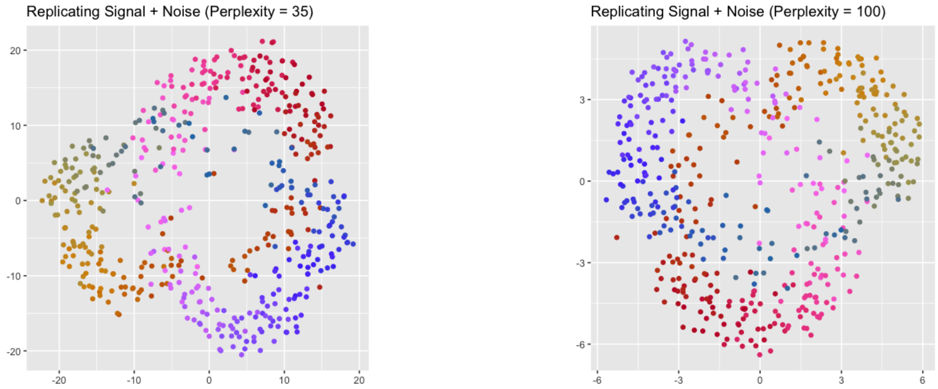

Fig B. Trustworthiness-maximizing representations for sd = 10 (trefoil). Trustworthiness-maximizing t-SNE outputs.

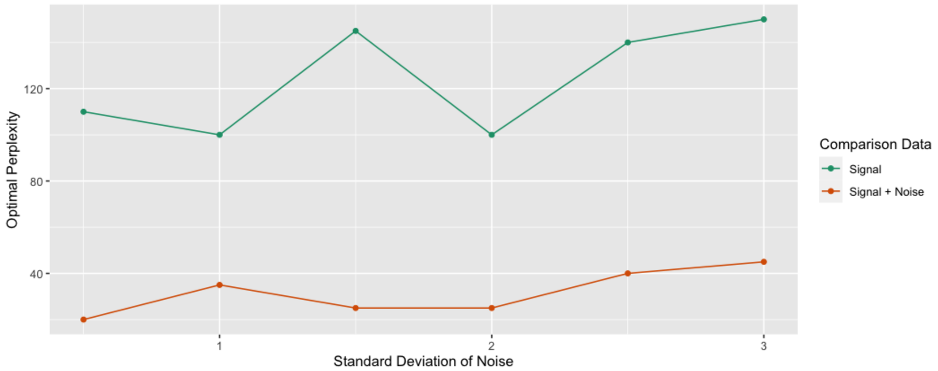

Fig C. Optimal perplexity (trefoil). The experiment was repeated at various levels of noise. For each level of noise, the trustworthiness-maximizing perplexity was recorded when comparing against the original data and the signal. The optimal perplexity was consistently greater when comparing against the signal.

1.2 Mammoth Plots

For the mammoth example, the signal Y consisted of 500 points in three dimensions. The data was randomly sampled from the mammoth data set used in [18]. $Z + \epsilon$ was constructed by adding seven superfluous dimensions and isotropic Gaussian noise. Various degrees of noise were tested ($sd = 0.5, 1, 1.5, 2, 2.5, 3$). The first two plots depict Trustworthiness vs. Perplexity and the trustworthiness-maximizing embeddings for the $sd = 1$ case. The third plot shows the trustworthiness-maximizing perplexity for the different degrees of noise.

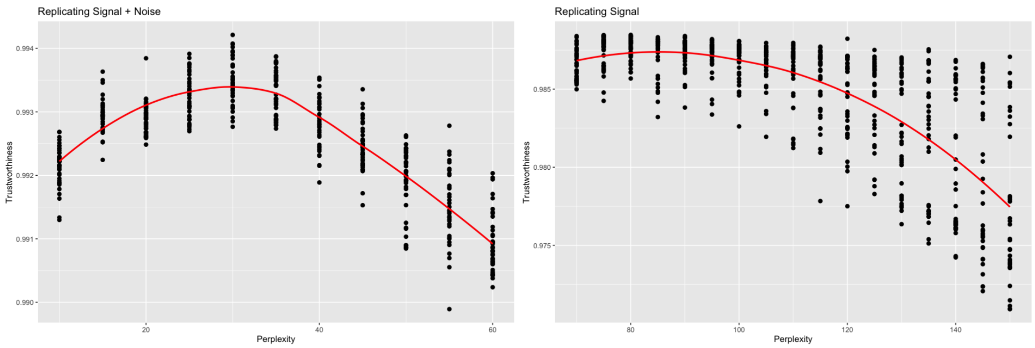

Fig D. Trustworthiness vs. perplexity for sd = 11 (mammoth). t-SNE outputs were calculated with varying perplexities. Local performance was measured via trustworthiness. The trustworthiness-maximizing perplexity was 30 when comparing against the original data, while the trustworthiness-maximizing perplexity was 80 when comparing against just the signal.

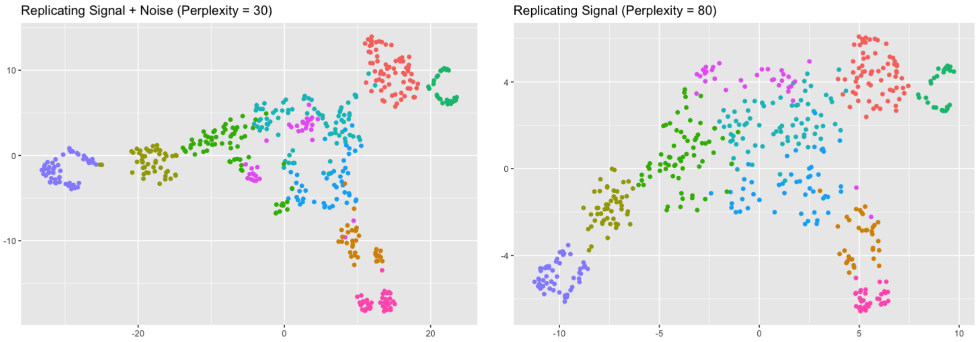

Fig E. Trustworthiness-maximizing representations (mammoth). Trustworthiness-maximizing t-SNE outputs. Labels provided by the authors of the data. The perplexity = 30 representation is more tightly clustered, but incorrectly separated the purple cluster, while the perplexity = 80 representation did not.

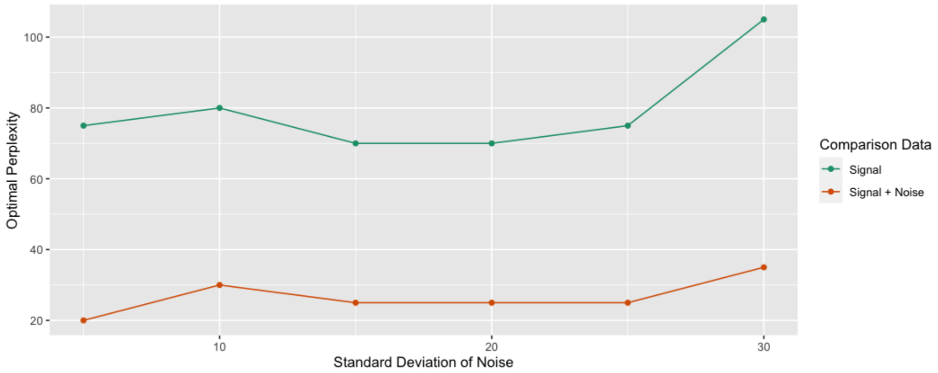

Fig F. Optimal perplexity (mammoth). The experiment was repeated at various levels of noise. For each level of noise, the trustworthiness-maximizing perplexity was recorded when comparing against the original data and the signal. The optimal perplexity was consistently greater when comparing against the signal.

2 Practical Examples

2.1 CyTOF Data Set

The CyTOF data set contained 239,933 observations in 49 dimensions [21]. To reduce the computational load, a subset of 5,000 observations was sampled. A log transformation was followed by a PCA pre-processing step to reduce the number of dimensions to 30, which still retained 77% of the variance in the original data. The processed data set to be studied consisted of 5,000 observations in 30 dimensions. The signal was first taken to be the first five principal components, then the first eight principal components. A hierarchical clustering of the high-dimensional data was computed then projected onto the trustworthiness-maximizing representations.

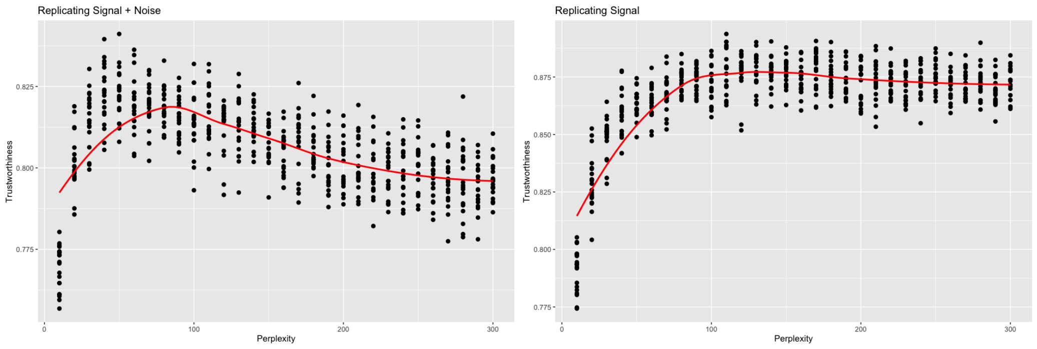

Fig G. Trustworthiness vs. perplexity for r = 5 (CyTOF). t-SNE outputs were calculated with varying perplexities. Local performance was measured via trustworthiness. The trustworthiness-maximizing perplexity was 50 when comparing against the original data, while the trustworthiness-maximizing perplexity was 110 when comparing against just the signal.

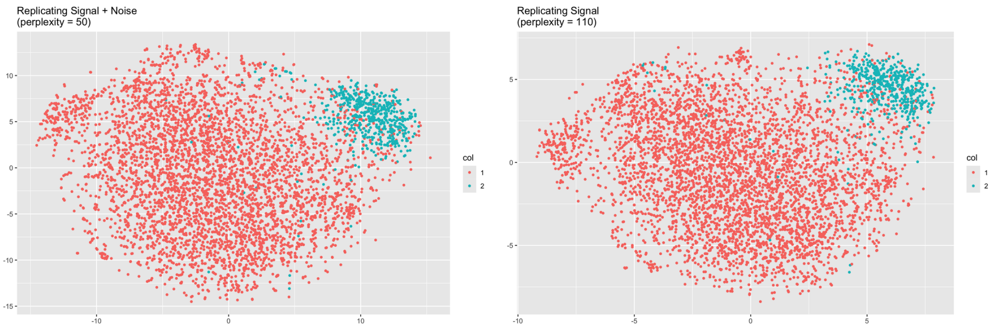

Fig H. Trustworthiness-maximizing representations for r = 5 (CyTOF). Trustworthiness-maximizing t-SNE outputs. Hierarchical clustering applied to high-dimensional data.

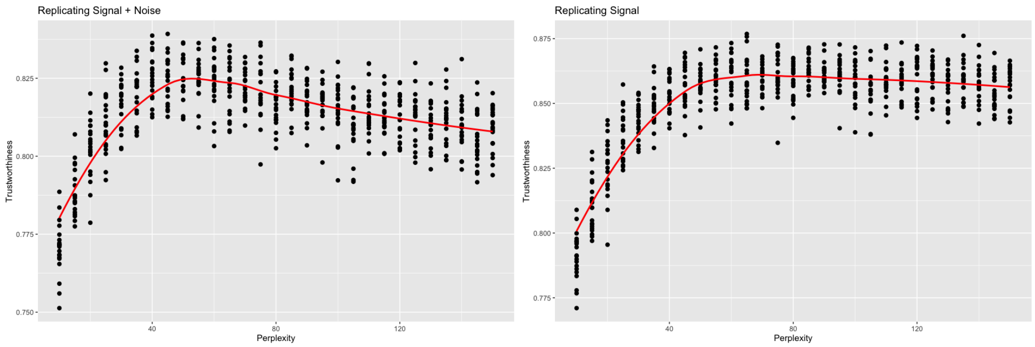

Fig I. Trustworthiness vs. perplexity for r = 8 (CyTOF). t-SNE outputs were calculated with varying perplexities. Local performance was measured via trustworthiness. The trustworthiness-maximizing perplexity was 45 when comparing against the original data, while the trustworthiness-maximizing perplexity was 65 when comparing against just the signal.

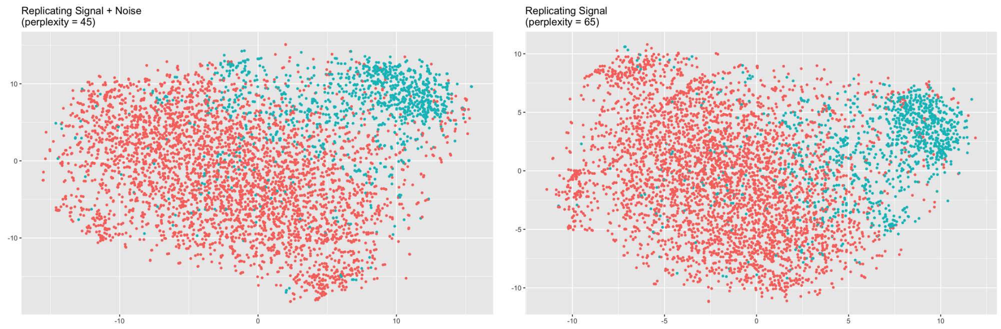

Fig J. Trustworthiness-maximizing representations for r = 8 (CyTOF). Trustworthiness-maximizing t-SNE outputs. Hierarchical clustering applied to high-dimensional data.

2.2 Microbiome Data Set

[22] compares the faecal microbial communities from 22 subjects using complete shotgun DNA sequencing. The original data contained 280 samples and 553 genera. To deal with a large number of near-zero readings, columns containing a large proportion of values less than 10^{-6} (60% or more) were removed. This reduced the dimension to 66. A PCA pre-processing was used to center and re-scale the data. The signal was first taken to be the first five principal components, then the first eight principal components. A k-means clustering of the high-dimensional data was computed then projected onto the trustworthiness-maximizing representations.

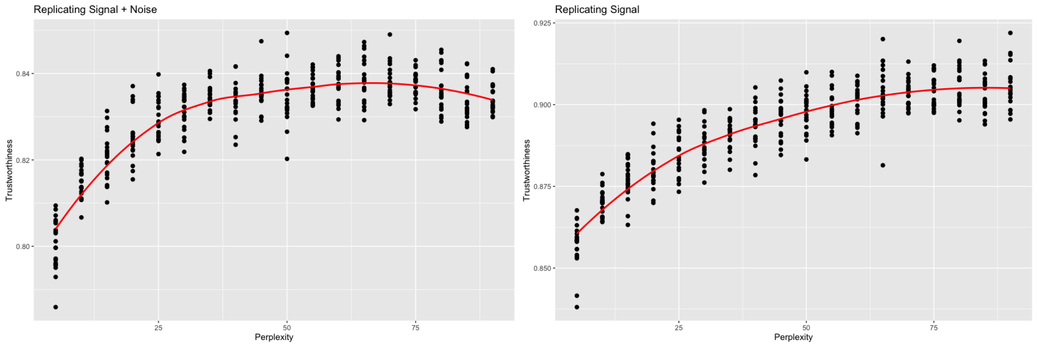

Fig K. Trustworthiness vs. perplexity for r = 5 (microbiome). t-SNE outputs were calculated with varying perplexities. Local performance was measured via trustworthiness. The trustworthiness-maximizing perplexity was 50 when comparing against the original data, while the trustworthiness-maximizing perplexity was 90 when comparing against just the signal.

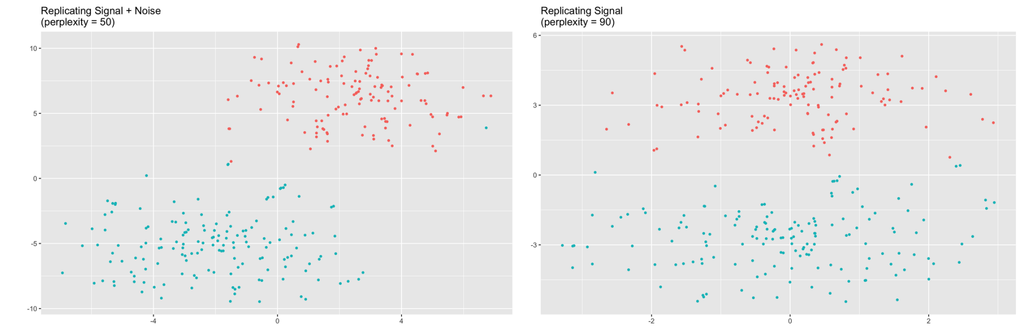

Fig L. Trustworthiness-maximizing representations for r = 5 (microbiome). Trustworthiness-maximizing t-SNE outputs. K-means clustering applied to high-dimensional data.

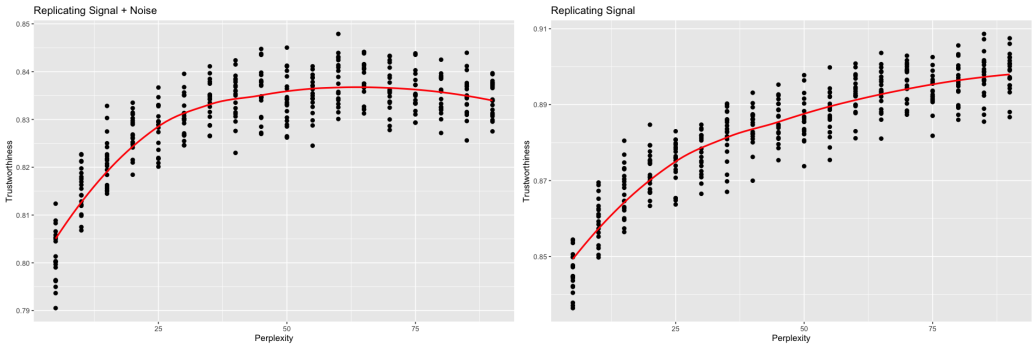

Fig M. Trustworthiness vs. perplexity for r = 8 (microbiome). t-SNE outputs were calculated with varying perplexities. Local performance was measured via trustworthiness. The trustworthiness-maximizing perplexity was 60 when comparing against the original data, while the trustworthiness-maximizing perplexity was 85 when comparing against just the signal.

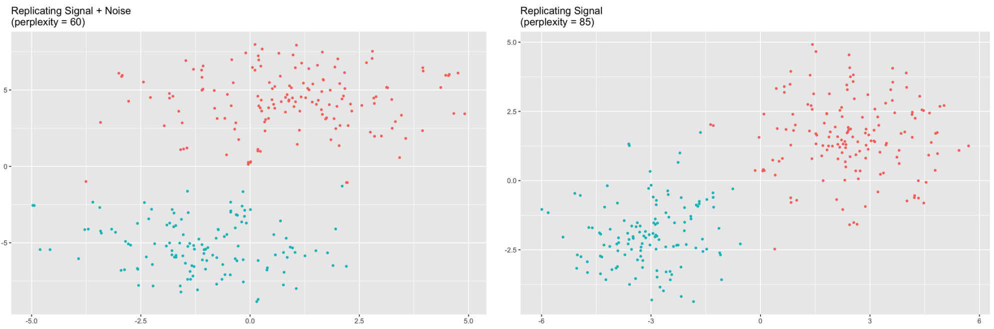

Fig N. Trustworthiness-maximizing representations for r = 8 (microbiome). Trustworthiness-maximizing t-SNE outputs. K-means clustering applied to high-dimensional data.

3 PBMC Data Set

Further examination of the dendritic cells shows that they belong to multiple clusters. To determine the number of clusters, we employ average silhouette width and the gap statistic.

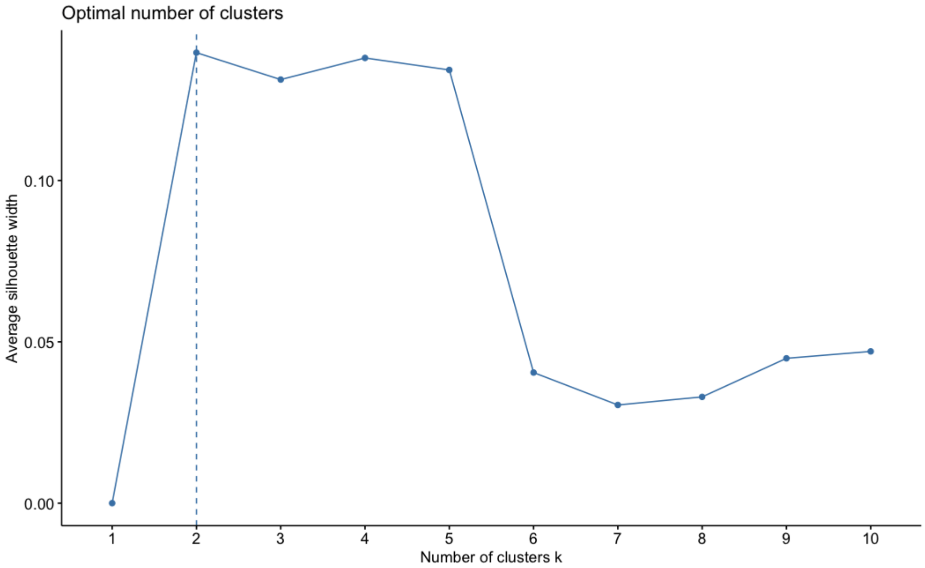

Fig O. Average silhouette width for dendritic cells. The average silhouette width suggests two clusters are appropriate.

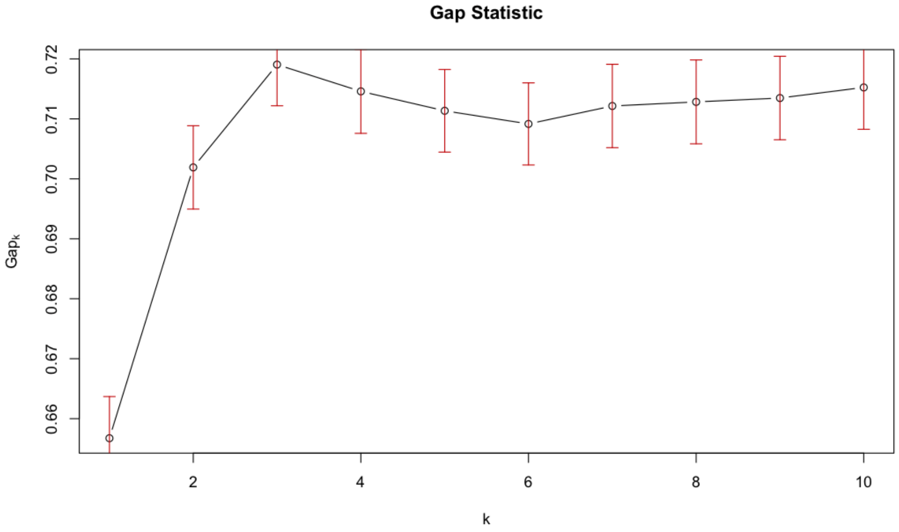

Fig P. Gap statistic for dendritic cells. The gap statistic suggests three clusters are appropriate.

References

[1] Amir ED, Davis KL, Tadmor MD, Simonds EF, Levine JH, Bendall SC, et al. viSNE enables visualization of high dimensional single-cell data and reveals phenotypic heterogeneity of leukemia. Nat Biotechnology. 2013 May 19;31:545-52.

[2] Alter O, Brown PO, Botstein D. Singular value decomposition for genome-wide expression data processing and modeling. PNAS. 2000 Aug 29;97(18):10101-6.

[3] Moon KR, van Dijk D, Wang Z, Gigante S, Burkhardt DB, Chen WS, et al. Visualizing structure and transitions in high-dimensional biological data. Nat Biotechnology. 2019 Dec 3;37:1482-92.

[4] van der Maaten L, Hinton G. Visualizing data using t-SNE. JLMR. 2008 Nov 8;9:2579-605.

[5] McInnes L, Healy J, Melville J. UMAP: Uniform Manifold Approximation and Projection for dimension reduction. arXiv:1802.03426v3 [Preprint]. 2020. Available from https://arxiv.org/abs/1802.03426.

[6] Becht E, McInnes L, Healy J, Dutertre CA, Kwok IWH, Ng LG, et al. Dimensionality reduction for visualizing single-cell data using UMAP. Nat Biotechnology. 2019 Dec 3;37:28-44.

[7] Kobak D, Linderman GC. Initialization is critical for preserving global data structure in both t-SNE and UMAP. Nat Biotechnology. 2021 Feb 1;39:156-7.

[8] Crecchi F, de Bodt C, Verleysen M, Lee JA, Bacciu D. Perplexity-free parametric t-SNE. arXiv:2010.01359v1 [Preprint]. 2020. Available from https://arxiv.org/abs/2010.01359.

[9] Huang H, Wang Y, Rudin C, Browne EP. Towards a comprehensive evaluation of dimension reduction methods for transcriptomic data visualization. Commun Biol. 2022 July 19;5(1):719.

[10] Kobak D, Berens P. The art of using t-SNE for single-cell transcriptomics. Nat Communications. 2019 Nov 28;10:5416.

[11] Cao Y, Wang L. Automatic selection of t-SNE perplexity. arXiv:1708.03229.v1 [Preprint]. 2017. Available from https://arxiv.org/abs/1708.03229.

[12] Coifman RR, Lagon S. Diffusion maps. Applied and Computational Harmonic Analysis. 2006 July;21(1):5-30.

[13] Wattenberg M, Viégas F, Johnson I. How to Use t-SNE Effectively. Distill. 2016. Available from https://distill.pub/2016/misread-tsne/.

[14] Coenen A, Pearce A for Google PAIR. Understanding UMAP. Available from https://pair-code.github.io/understanding-umap/.

[15] Chari T, Pachter L. The specious art of single-cell genomics. PLoS Computational Biology. 2017 Aug 17;19(8): e1011288.

[16] Espadoto M, Martins RM, Kerren A, Hirata NST, Telea AC. Towards a quantitative survey of dimension reduction techniques. IEEE Transactions on Visualization and Computer Graphics. 2021 Mar;27(3):2153-73.

[17] Venna J, Kaski S. Visualizing gene interaction graphs with local multidimensional scaling. ESANN. 2006 Apr 26-28.

[18] Wang Y, Huang H, Rudin C, Shaposhnik Y. Understanding how dimension reduction tools work: An empirical approach to deciphering t-SNE, UMAP, TriMap, and PaCMAP for data visualization. JMLR. 2021;22:1-73.

[19] Krijthe JH. Rtsne: T-Distributed Stochastic Neighbor Embedding using a Barnes-Hut implementation. Available from https://github.com/jkrijthe/Rtsne.

[20] Tung PY, Blischak JD, Hsiao CJ, Knowles DA, Burnett JE, Pritchard JK, et al. Batch effects and the effective design of single-cell gene expression studies. Scientific Reports. 2017 Jan 3;7:39921.

[21] Strauss-Albee DM, Fukuyama J, Liang EC, Yao Y, Jarrell JA, Drake AL, et al. Human NK cell repertoire diversity reflects immune experience and correlates with viral susceptibility. Science Translational Medicine. 2015 July 22;7(297):297.

[22] Arumugam M, Raes J, Pelletier E, Paslier DL, Yamada T, Mende DR, et al. Enterotypes of the human gut microbiome. Nature. 2011 Apr 20;473:174-80.

[23] Horn JL. A rationale and test for the number of factors in factor analysis. Psychometrika. 1965 June;30:179-85.

[24] Skrodzki M, Chaves-de-Plaza N, Hildebrandt K, Höllt T, Eisemann E. Tuning the perplexity for and computing sampling-based t-SNE embeddings. arXiv:2308.15513 [Preprint]. 2023 Aug 29. Available from https://arxiv.org/abs/2308.15513.

[25] Cell Ranger ARC 2.0.0. PBMC from a healthy donor - granulocytes removed through cell sorting (3k). 10x Genomics. 2021 May 5.

[26] Parks B. BPCells: single cell counts matrices to PCA. 2024. Available from https://bnprks.github.io/BPCells.

[27] Chu SK, Zhao S, Shyr Y, and Liu Q. Comprehensive evaluation of noise reduction methods for single-cell RNA sequencing data. Briefings in Bioinformatics. 2022 Mar 10;23(2).

[28] Amid E, Warmuth MK. TriMap: Large-scale dimensionality reduction using triplets. arXiv:1910.00204.v2 [Preprint]. 2022 Mar 26. Available from https://arxiv.org/abs/2308.15513.

[29] Lee JA, Verleysen M. Quality assessment of dimensionality reduction: Rank-based criteria. Neurocomputing. 2009 Mar;72(7-9):1431-43.

[30] Schreck T, von Landesberger T, Bremm S. Techniques for precision-based visual analysis of projected data. Sage. 2012 Jan 1;9(3).
