# Supplementary material for: Calibrating dimension reduction hyperparameters in the presence of noise
Source: PLoS Comput Biol. 2024 Sep 12;20(9):e1012427. doi: 10.1371/journal.pcbi.1012427 (PMC11421778; doi:10.1371/journal.pcbi.1012427)
Supplement: Attachment — Submitted filename: resubmission letter.docx [file pcbi.1012427.s002.docx]

Dear editors and reviewers,

Thank you for the constructive feedback regarding our manuscript, *Calibrating dimension reduction hyperparameters in the presence of noise*. We’ve incorporated much of the feedback to create a new, heavily revised version. In this letter, we describe the major changes and respond to the reviewers’ comments.

The major adjustments to the manuscript include additions to the pre-existing experiments and the addition of a case study. As per Reviewer #1’s suggestion, we’ve demonstrated how the optimal perplexity changes as the magnitude of noise increases via Optimal Perplexity vs. Level of Noise plots for the simulated examples. We also included visualizations of the trustworthiness-maximizing representations to illustrate the effects of hyperparameter re-calibration as Reviewer #2 suggested. The last major change was the addition of a case study demonstrating how to use the framework in practice as Reviewer #1 suggested. The new section applies the framework step-by-step to an application of UMAP to a modern 10x Genomics dataset. The results are then compared to demonstrate how our hyperparameter calibration strategy affects the result.

We now respond to the reviewers’ comments.

**Reviewer #1:**

1) “For simulated examples, it would be useful to sweep over signal-to-noise ratio (by adjusting the gaussian noise) and plotting the optimal perplexity as the SNR changes. This can also be performed for practical examples by sweeping over PCs. E.g. I would recommend keeping one of Fig 6/7 and add a plot that shows the optimal perplexity as a function of PCs.”

- We included these plots for the simulated examples. However, we did not for the practical examples because the signal-to-noise ratio should be an inherent property of the data set. It is not meant to “vary”. With the simulated examples, we can adjust the level of noise to create new data sets that should be treated independently.

2) “My recommendation would be to add a detailed case-study that applies the authors proposed recommendations (in Section 5) and walks the reader through a quantitative and qualitative evaluation of the results.”

- This has been added to the manuscript.

3) All of the minor comments were addressed and fixed.

**Reviewer #2:**

1) “First, while t-SNE and UMAP do not consider the noise in the data, many other methods do. In particular, methods based on the diffusion maps framework denoise the data as part of the dimensionality reduction. These methods include, but are not limited to, diffusion maps [R1], PHATE [R2], MultiScale PHATE [R3], RF-PHATE [R4], EIG [R5], and DIG [R6]. The amount of diffusion is often considered in the context of denoising as well (see [R2]). So these references should be included and the authors' claims that noise is not considered in dimensionality reduction should be softened, if not eliminated entirely. This diminishes the claimed novelty of the proposed work.”

- We have included these references and a discussion on the role hyperparameters play in de-noising data during dimension reduction. We’ve also re-worded the thesis of our manuscript. Our goal is to show t-SNE and UMAP, the two most popular nonlinear dimension reduction methods, can be calibrated to handle noise well like modern DR techniques that also rely on hyperparameter calibration for de-noising. PHATE, for example, requires calibration of “diffusion time scale” t, which acts as a smoothing agent and balances attention to local and global aspects of the data. Perplexity and n_neighbors serve the same purpose, but their typical calibration processes rarely consider noise, while PHATE’s does. We show the typical, naïve processes for t-SNE/UMAP hyperparameter calibration are subpar and lead to overfitting.

2) “Based on the authors' results, they do give different guidelines for hyperparameters for t-SNE and UMAP than is commonly suggested. These are obtained by effectively optimizing the trustworthiness and/or Shepard goodness with respect to the PCA representation. However, the authors never show any actual t-SNE or UMAP visualizations that compare the different hyperparameters. Thus it is not clear if the new suggested values offer any improvement over the old ones from a visual perspective.”

- Trustworthiness-maximizing representations have been added for the cases in which the signal structure is well understood. We show the new hyperparameter values also increase qualitative performance.

3) “Section 3.3: Modeling the embedding function using the PCA inverse transform seems very limiting and also seems to undermine the proposed approach as the optimal dimensionality reduction in this setting would be to do PCA. It's not clear why anyone would want to do t-SNE or UMAP to reduce dimensions if PCA is the optimal embedding function. It seems we would need more complex embedding functions to justify the use of t-SNE or UMAP (or anything besides PCA). Because of this potential disparity, it is not clear that using PCA to represent the "true" signal would correlate well with good hyperparameters for methods like t-SNE or UMAP. Hence the need for visualizations to verify this, especially in the case for the simulated and simple datasets.”

- We believe it is important to distinguish between visually representing data and quantitatively representing data. The PCA projection is meant to represent quantitative aspects of the data, i.e. nearest neighbors, inter-point similarities, global positioning of clusters, etc. Such an embedding is quantitatively faithful, but not necessarily the best visual representation. t-SNE and UMAP are meant to visually represent data by stretching and contracting space in a way that exaggerates certain patterns. While such an embedding may be quantitatively misrepresentative of the data (cluster sizes, distances between clusters, etc.), it reveals important patterns of interest. The goal of our framework is to create useful visualizations via t-SNE/UMAP that are more quantitatively faithful to the signal. As such, we are interested in relative trustworthiness, or trustworthiness-maximizing t-SNE/UMAP outputs, rather than the actual trustworthiness value. The additional visualizations also more support for our framework.

4) “Given this is PLOS Computational Biology, the introduction should talk more about biology uses for visualization than it currently does. In addition to the references above, the authors should reference [R7-R9, etc.].”

- We’ve added more discussion in the introduction.

5) “Line 66: many other downsides of t-SNE exist that should be discussed. See [R10]. UMAP inherits many of these as well.”

- We’ve added mentions of the other downsides of t-SNE/UMAP.

6) “Section 2.2: It has been shown that the main benefit that UMAP has over t-SNE is its initialization using Laplacian eigenmaps. Initializing t-SNE with Laplacian eigenmaps gives similar results to UMAP [R9]. This is worth mentioning. Perhaps it is worth exploring if the recommendations change when t-SNE is initialized using Laplacian eigenmaps instead of the default random initialization.”

- We’ve added more discussion regarding t-SNE vs. UMAP and their similarities/dissimilarities.

7) “A better alternative to the Shepard goodness is the Mantel test. The Mantel test takes into account the correlations between distances, which is largely ignored in the Shepard goodness.”

- The Mantel test applies Pearson correlation, a measure of linear correlation, and Shepard goodness applies Spearman correlation, a rank-based correlation. We believe a rank-based correlation is more appropriate here because of the nonlinearity of t-SNE/UMAP. These algorithms stretch and shrink space non-uniformly, so the inter-point distances will not be affected uniformly. As such, the inter-point distances in high and low dimensions will not be, and probably shouldn’t be, linearly correlated. Global positioning should instead be represented by the ranking of inter-point distances.

8) “Section 4.1: How are the # of PCA dimensions chosen for these experiments?”

- For the simulated examples, the signal dimension is known. For example, the links example was constructed from a signal of two inter-locked circles embedded in three dimensions, so the signal is three-dimensional. For the practical examples, the number of signal dimensions is chosen using scree plots.

Justin Lin

Indiana University Bloomington

[linjus@iu.edu](mailto:linjus@iu.edu)

Julia Fukuyama

Indiana University Bloomington

[jfukuyam@iu.edu](mailto:jfukuyam@iu.edu)
