# Supplementary material for: Calibrating dimension reduction hyperparameters in the presence of noise
Source: PLoS Comput Biol. 2024 Sep 12;20(9):e1012427. doi: 10.1371/journal.pcbi.1012427 (PMC11421778; doi:10.1371/journal.pcbi.1012427)
Supplement: Attachment — Submitted filename: resubmission_letter_v2.docx [file pcbi.1012427.s003.docx]

Dear editors and reviewers,

Thank you for the constructive feedback regarding our manuscript, *Calibrating dimension reduction hyperparameters in the presence of noise*. We’ve incorporated the feedback to create a new, revised version. In this letter, we address the comments and describe the revisions.

Reviewer #1: Comments satisfactorily addressed in previous revision.

Reviewer #2:

1) “The main thing I would still like to see is t-SNE visualizations for the datasets used in Section 4.2.”

We’ve have added visualizations for the practical examples to illustrate the effects of increasing perplexity. We also chose to switch out the main practical example in the manuscript with one from the Supporting Information. More specifically, the scRNA-seq example is now included in the main manuscript, while the CyTOF example was moved to the Supporting Information.

We’ve chosen to make this switch because the CyTOF example is visually less interesting. The data contain two clusters, so the inter-cluster relationships and cluster positioning are rather arbitrary. The scRNA-seq data contain more clusters, making better discussion of perplexity’s effect on inter-cluster relationships. This example better illustrates the effect of increasing perplexity.

2) “Figure 18 appears to be miscaptioned and could probably be combined with Figure 16.”

The caption for Figure 18 has been fixed.

Thank you for your time,

Justin Lin

Indiana University Bloomington

[linjus@iu.edu](mailto:linjus@iu.edu)

Julia Fukuyama

Indiana University Bloomington

[jfukuyam@iu.edu](mailto:jfukuyam@iu.edu)
