# Supplementary material for: Calibrating dimension reduction hyperparameters in the presence of noise
Source: PLoS Comput Biol. 2024 Sep 12;20(9):e1012427. doi: 10.1371/journal.pcbi.1012427 (PMC11421778; doi:10.1371/journal.pcbi.1012427)
Supplement: Attachment — Submitted filename: resubmission_letter_v3.docx [file pcbi.1012427.s004.docx]

Dear editors and reviewers,

We dearly thank you for your time and wisdom when reviewing our manuscript, *Calibrating dimension reduction hyperparameters in the presence of noise*. The current iteration is a vast improvement upon the first draft submitted last year, thanks to your help. We have incorporated this round of feedback, and the adjustments are outlined here.

Reviewer #2:

1) “On page 11, the discussion of Figure 11 discusses the relative position of clusters and how that changes with different values of perplexity. An important point is that the relative position of clusters in t-SNE embeddings is arbitrary and meaningless. Thus the authors should either eliminate this discussion or clarify this point.”

You are correct that relative positioning of clusters in t-SNE embeddings is often misleading, so we’ve backed up the claim with additional evidence in the form of Shepard goodness. The increased perplexity leads to a significant increase in Shepard goodness, implying the re-positioning of clusters was both meaningful and beneficial.

Thank you for your time,

Justin Lin

Indiana University Bloomington

[linjus@iu.edu](mailto:linjus@iu.edu)

Julia Fukuyama

Indiana University Bloomington

[jfukuyam@iu.edu](mailto:jfukuyam@iu.edu)
